# Effects of Large-Scale Municipal Safe Routes to School Infrastructure on Student Active Travel and Physical Activity: Design, Methods, and Baseline Data of the Safe Travel Environment Evaluation in Texas Schools (STREETS) Natural Experiment

**DOI:** 10.3390/ijerph19031810

**Published:** 2022-02-05

**Authors:** Deanna M. Hoelscher, Leigh Ann Ganzar, Deborah Salvo, Harold W. Kohl, Adriana Pérez, Henry Shelton Brown, Sarah S. Bentley, Erin E. Dooley, Amir Emamian, Casey P. Durand

**Affiliations:** 1 Michael and Susan Dell Center for Healthy Living, School of Public Health in Austin, The University of Texas Health Science Center at Houston (UTHealth), Austin, TX 78701, USA; leigh.a.ganzar@uth.tmc.edu (L.A.G.); harold.w.kohl@uth.tmc.edu (H.W.K.III); adriana.perez@uth.tmc.edu (A.P.); henry.s.brown@uth.tmc.edu (H.S.B.); sarah.s.bentley@uth.tmc.edu (S.S.B.); 2 Prevention Research Center in St. Louis, Brown School, Washington University, St. Louis, MO 63130, USA; dsalvo@wustl.edu; 3 Department of Kinesiology and Health Education, The University of Texas at Austin, Austin, TX 78712, USA; 4 Department of Epidemiology, School of Public Health, University of Alabama at Birmingham, Birmingham, AL 35233, USA; edooley@uab.edu; 5 Public Works Department, City of Austin, Austin, TX 78704, USA; amir.emamian@austintexas.gov; 6 Michael and Susan Dell Center for Healthy Living, Department of Health Promotion & Behavioral Sciences, School of Public Health in Houston, The University of Texas Health Science Center at Houston, Houston, TX 77030, USA; casey.p.durand@uth.tmc.edu

**Keywords:** Safe Routes to School, physical activity, children, active commuting, natural experiment

## Abstract

Past evaluations of Safe Routes to School (SRTS) programs have been relatively small in scope and have lacked objective measurements of physical activity. A 2016 Mobility Bond in Austin, Texas, USA, allocated USD 27.5 million for infrastructure changes to facilitate active commuting to schools (ACS). The Safe TRavel Environment Evaluation in Texas Schools (STREETS) study aims to determine the health effects of these infrastructure changes. The purpose of this paper is to describe the STREETS study design, methods, and selected baseline results. The STREETS study is comprised of two designs: (1) a serial cross-sectional design to assess changes in ACS prevalence, and (2) a quasi-experimental, prospective cohort to examine changes in physical activity. Differences between study arms (Austin SRTS and comparison) were assessed for school demographics, ACS, and school programs. At baseline, 14.3% of school trips were made by ACS, with non-significant differences between study arms. Only 26% of schools implemented ACS-related programs. Some significant differences across SRTS and comparison schools were identified for several school- and neighborhood-level characteristics. Substantial changes are needed across area schools and neighborhoods to promote optimum ACS. STREETS study longitudinal findings will be critical for informing optimal future implementations of SRTS programs.

## 1. Introduction

Physical activity in children has been linked to numerous physical health benefits, including weight control, musculoskeletal health and fitness, cardiovascular health, cognitive health, and functioning [1,2,3]. Active commuting to and from school (ACS) is a meaningful source of physical activity in children and has the potential to contribute substantially to helping children meet the physical activity guidelines for Americans goal of 60 min of moderate to vigorous intensity physical activity (MVPA) per day [4,5]. Children who engage in ACS are more physically active than children who commute by vehicle [6,7]. For example, a meta-analysis was used to analyze twelve studies with objectively measured physical activity and found the weighted mean MVPA for those that engage in ACS was 17 min per day among elementary school students, representing 23% of total MVPA per day for those children [8].

Despite the health benefits of ACS [9], walking or biking to school has decreased over time; an estimated 40.7 percent of students walked or biked to school in 1969, but by 2001, that number dropped to 12.9 percent [10]. More recent data, from 2017, show that 9.6% of school-aged children walk to school on a regular basis, while an additional 1.1% bicycle [11]. In the face of this decline, there is growing evidence of the importance of the built environment for promoting ACS [12,13]. The American Heart Association’s recent policy on the built environment and active travel highlights the importance of mesoscale policies that improve pedestrian and bicycle networks and infrastructure for health [14] Built environment interventions, especially those with planned links to physical activity outcomes, are relatively rare compared to educational interventions due to cost of implementation and ease of evaluation [15].

Safe Routes to School (SRTS) became federal policy in the United States in 2005 with a transportation bill [16] aiming to create safe, convenient, and fun opportunities for children to bicycle or walk to and from school and include a built environment transformation component. SRTS initiatives seek to stimulate community, health, and academic improvements through the six E’s: engagement, equity, engineering, encouragement, education, and evaluation [17]. SRTS engineering projects have included infrastructure changes in the built environment around schools to increase ACS, and funds have been allocated from a diverse range of sources, including federal, local and private ones [18]. However, to date, the majority of SRTS evaluations have narrowly assessed the impacts of education or encouragement interventions on ACS [19,20,21,22]. The National Center for Safe Routes to School report on SRTS programs and environmental changes from 2007 to 2014, found that the prevalence of walking to school increased from 11.9% to 15.2% for morning arrival to school, and from 15.2% to 18.4% for departures from school, with higher increases among children attending low-income schools [23].

To date, evaluations of SRTS interventions with built environment engineering or infrastructure changes have suffered from inadequate trials and a lack of objectively measured physical activity [24,25]. A study by McDonald et al. [26] found that engineering improvements were associated with an 18% increase in walking and biking to school in schools with SRTS funding, compared to similar schools with no SRTS programs. In a longitudinal study spanning 12 years, Chen et al. [27] found that sidewalk and bike lane improvements resulted in an increase in the number of students who engage in ACS. Unfortunately, project engineering costs and objectively measured MVPA were not assessed in either of these two studies. Another study by Hoelscher et al. [28] found modest short-term increases in ACS as a result of SRTS interventions, with few differences between large infrastructure interventions (e.g., engineering changes in the built environment) versus non-infrastructure interventions (e.g., SRTS plans or programs). However, qualitative data suggest poor implementation of SRTS infrastructure projects or programs, reinforcing the need for greater project management and oversight [29].

To strengthen the evidence for the positive health impacts of infrastructure changes, the *Research Roadmap for Transportation and Public Health* identified several needs for research in the United States, including “…population level natural experimental/observational studies on the economic impacts (e.g., transportation and health costs) of transportation policy changes intended to support health” [30]. In addition, studies have recommended that local planners pursue federal, state, and local funds for infrastructure improvements, including capital improvement planning and budgeting as a potential source of funds for SRTS programs [31,32].

In 2016, citizens of Austin, TX, USA approved the Austin Mobility Bond, which allocated USD 720 million in funding to transportation and mobility improvements at the local, corridor, and regional levels. Of these funds, USD 27.5 million was allocated to SRTS infrastructure projects, with additional funds designated to bikeways, sidewalks, urban trails, fatality reduction strategies, and substandard streets/capital renewal [33]. Municipal funding of SRTS programs by local bonds can offer a feasible means of ensuring stronger project oversight, management, and implementation, and allow for robust measurement of the health effects of this funding, hence addressing a critical gap in the literature.

The purpose of this paper is to describe the design and methods of the Safe TRavel Environment Evaluation in Texas Schools (STREETS) study, an ongoing (2018–2023), five-year natural experiment to evaluate the health and behavioral impacts of the City of Austin’s SRTS projects, and to present baseline results from the serial cross-sectional study. The primary objective of this natural experiment is to evaluate the effects of the Austin Safe Route to School infrastructure projects on the prevalence of ACS, as well as on child-level physical activity outcomes. Since this is a natural experiment, the intervention was developed and is being implemented by city government with no health evaluation; our study team is evaluating the health outcomes of this program [34]. The overarching hypothesis of the STREETS study is that students enrolled in schools that participate in the Austin SRTS infrastructure projects will engage in more ACS and will accrue more overall MVPA compared to students in schools without infrastructure funding.

## 2. Materials and Methods

### 2.1. Overall Study Aims and Hypotheses

The STREETS study is powered to address two hypotheses: (1) schools with implementation of SRTS infrastructure projects will have at least a 7% mean increase in the proportion of students in third through fifth grades (8–11 years old) that engage in ACS versus schools without SRTS infrastructure projects (i.e., we will assess mean changes in ACS prevalence); and (2) students in third through fifth grades (8–11 years old) in schools with implementation of SRTS infrastructure projects will engage in a minimum detectable difference of at least five more minutes of MVPA per day compared to students enrolled in schools without infrastructure funding (i.e., we will assess individual-level changes in MVPA). STREETS will also examine the cost-effectiveness of SRTS infrastructure changes on child physical activity.

### 2.2. Study Design

**Figure 1:**
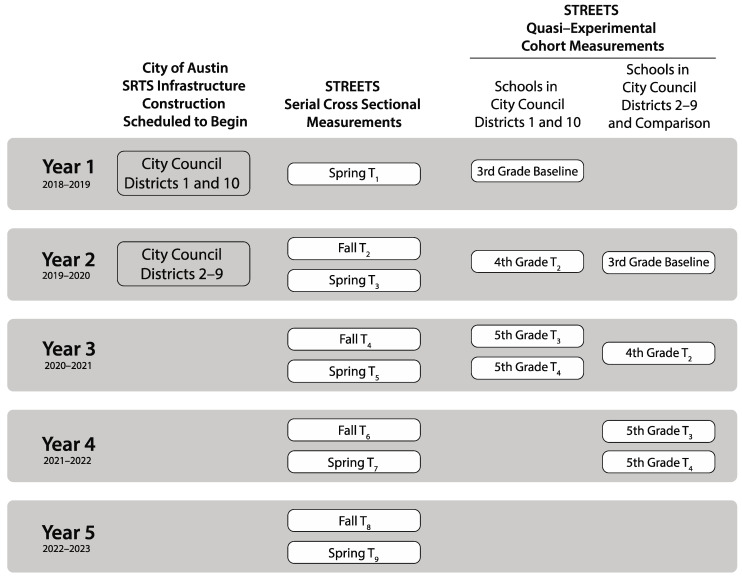
The Safe TRavel Environment Evaluation in Texas Schools (STREETS) Study Design and Measurement Schedule.

To examine the effects of SRTS infrastructure on changes in prevalence of ACS, the STREETS study uses a five-year, serial cross sectional study design (Figure 1). Each semester (spring/fall), modes of commuting to school are measured among students in third, fourth, and fifth grade enrolled in schools participating in the Austin SRTS infrastructure projects and comparison schools (no infrastructure projects) using a standard ACS tally count (self-report) [35].

For the second hypothesis, examining the effect of SRTS infrastructure on individual-level changes in daily MVPA among children, we use a quasi-experimental school cohort study design (Figure 1). Third grade students and their parents are being recruited from a randomly selected subsample of Austin schools and comparison schools, and these child-parent dyads will be followed for three years using accelerometers, GPS devices, and survey tools. The timing of recruitment is on a rolling basis in the cohort study, dependent upon the City of Austin’s infrastructure building schedule—cohort schools are being enrolled immediately prior to construction of the infrastructure projects, with the intent that infrastructure projects are completed and in use before final measurements when children are in fifth grade. After four measurements, accelerometer data collected from the cohort study and SRTS infrastructure project costs from the City of Austin will be used to assess relative return on investment. Baseline data for the cohort arm are not complete, as STREETS is currently recruiting participants as part of the on-going study enrollment (Figure 1).

### 2.3. Theoretical Framework

The STREETS evaluation framework is based on the social–ecological model, as interpreted by Perry et al. [36] for children. This model posits that an individual’s behavior is influenced by proximal and distal, and social and physical environments, such as SRTS infrastructure changes [37,38]. In addition, this iteration of the model draws on social cognitive theory to provide added interpretation of the factors that influence behaviors in children and adolescents, and we incorporate constructs such as self-efficacy and outcome expectations in our measures as psychosocial short-term outcomes [39,40]. Our study constructs and measures address several levels of the model, from intra- and interpersonal measures to organizational policies, to the community and built environment levels. The collection of data at various levels allows for investigation of system-level changes. This approach allows for assessment of major outcomes as well as the ability to determine other related behavioral changes and unintended consequences. By conducting both parent- and child-surveys, data at the interpersonal and intrapersonal levels, respectively, are available. At the organizational policy level, process data on resource allocation and relevant school programs and policies (such as current SRTS education curricula implemented in both infrastructure and non-infrastructure schools) are being collected. Finally, at the community/school/physical environment level, opportunities for and safety around ACS, school policies, social capital, and built environment factors are being examined.

### 2.4. Natural Experiment: Intervention Description

In 2016, voters in Austin, Texas approved a municipal bond that allocated USD 720 million to transportation improvement projects, with USD 27.5 million dedicated to the Public Works Department for SRTS Infrastructure projects across the 280 square miles of the City of Austin jurisdiction [41]. Despite the significant funding allocated for this program, the city did not have plans for evaluation of health effects in school children, which provided an opportunity to conduct a natural experiment [34]. The funding was divided equally across 10 City Council Districts for projects at elementary and middle schools. A three-step process was used to determine which projects would be funded through the bond, which included walk audits, public engagement, and prioritization of proposed projects [42]. The City of Austin conducted walk audits at 110 Austin elementary and 27 Austin middle schools to assess the existing infrastructure within a half-mile radius for pedestrian facility recommendations and up to a two-mile radius for bicycle facilities around each school and identify barriers to safe walking and biking. The issues identified through the walk audits were supplemented by input from community members at in-person meetings and through online mapping platforms. Infrastructure projects were prioritized for construction based on both overall benefit and cost-benefit by scoring each project on demand, safety, equity, and stakeholder input. The prioritized projects range in scope, and include curb ramps, sidewalks, pedestrian hybrid beacons, pedestrian crosswalks, and bike lanes. More detailed information on the scoring and prioritization process is presented elsewhere [43]. Construction on the prioritized infrastructure projects began in 2018 and is scheduled to run through at least 2023, and as of January 2022, 264 SRTS projects have been completed, 3 projects are currently in construction, and 253 projects are in development [44].

The specific characteristics of the planned implementation of this SRTS effort in Austin, Texas, make it uniquely relevant for conducting a rigorous natural experiment because exposure to these infrastructure changes is not being manipulated by the evaluators, but the independent results can provide evidence of the health impacts of transportation planning and construction [34,45]. The types of proposed environmental changes are transformative and large in scale, and thus not limited to construction of basic, small cost projects (e.g., traffic calming, sidewalks, etc.) that lead to smaller changes in ACS. The Austin SRTS initiative provides a wider and more comprehensive range of large-scale engineering projects, such as protected bike lanes, shared use paths coordinated with other city departments/groups, pedestrian islands, and pedestrian hybrid beacons. This initiative is also designed to allocate funding based not only on the usual parameters of safety, cost and feasibility, but also factors health equity (the sixth ‘E’ of SRTS), ultimate impact, and community input into the prioritization of infrastructure projects.

### 2.5. Study Participants and Inclusion Criteria

#### 2.5.1. Serial Cross Sectional Study (Hypothesis 1)

Out of 110 elementary schools within the Austin city limits eligible for infrastructure changes, the STREETS study aimed to recruit 70 Austin SRTS infrastructure schools for the serial cross sectional study to address hypothesis 1. Additionally, the study aimed to recruit 30 comparison schools. To determine sample size, a priori power calculations assumed a 10% difference in the mean of the proportion of students in third through fifth grades that used active travel modes between both groups (Austin SRTS versus comparison), a two-sided type I error of 0.05, standard deviations of the difference in the mean proportions of 0.09, and a differential rate of drop out at 12% for schools [46]. Using these parameters, we estimated power between 0.99 and 0.91.

Recruitment in the serial cross sectional study has been completed, and study participants include third–fifth grade students (ages 8–11 years) enrolled at SRTS infrastructure schools and non-infrastructure comparison elementary schools. Inclusion criteria for participating schools included: having grades 3 through 5 on campus, capacity to increase ACS (e.g., no impediments to infrastructure changes), and having minimal transfer (student attending schools outside of their assigned catchment area) or bussed students. Informed consent procedures for principals and classroom teachers and study protocol were approved by the institutional review board (IRB) at The University of Texas Health Science Center at Houston (UTHealth) (HSC-SPH-17-0638) and by the research departments at each participating school district.

#### 2.5.2. Quasi-Experimental Cohort Study (Hypothesis 2)

Recruitment of the parent–child dyads for the cohort study is based on a priori power estimations that used a two-sided type I error level of 0.05 and the following assumptions: (i) a random selection of 15 students in third grade per each school as the homogenous cluster size; (ii) coefficients of variations of the cluster sizes from 0.4 to 0.8; (iii) subject-to-subject standard deviations from 7 to 10.5 min of MVPA; and (iv) several intra-cluster correlation coefficients (ICC) from 0.03 to 0.1 [47,48]. The unit of analysis is the school with children nested within for this quasi-experimental cohort, so we aim to recruit 30 randomly selected SRTS infrastructure schools and 15 randomly selected comparison schools from the population that are already participating in the serial cross sectional study.

Recruitment in the cohort study is ongoing, and inclusion criteria for the cohort parent–child dyads are: (a) child enrollment in the third grade at one of the selected schools; (b) living within one Euclidean mile of school (an approximately 20 min walk); (c) ability to engage in regular physical activity; and (d) parent and child ability to complete a survey in English or Spanish. Once schools were recruited, parent–child dyads were recruited through flyers sent home in school folders; presentations at PTA meetings or school fitness nights; and on-site information meetings. All parents provided informed consent for themselves and their children to participate in the cohort study, and children also provided written assent.

### 2.6. Measures

Study measures include school-based measurements, individual student-level and parent-level assessments, community/built environment measures, qualitative data to provide context to the study, and process evaluation data for assessing implementation of the Austin SRTS program (Table 1).

#### 2.6.1. Measures: Serial Cross Sectional Study

To determine percentage of student trips to/from school made by active travel modes, the standard SRTS student tally method is used [35,49], in which students in a classroom are asked about transport to/from school and indicate their travel mode by a show of hands which of the following travel modes they used to get to school and how they plan to get home from school: walk, bike, bus, family vehicle, carpool, public transit, or other. Three days of data are collected from all SRTS infrastructure and non-infrastructure schools in the cross-sectional study each fall and spring semester. To account for potential effects of weather conditions on ACS, weather data, such as temperature and precipitation, are collected from National Oceanic and Atmospheric Administration (NOAA) weather stations in Austin during morning and afternoon commute time periods during the data collection dates, utilizing a previous standardized protocol [28].

Data on school policies, other SRTS or related programs that might affect child physical activity, and health education programs are collected at all study schools once per school year. The school health policy survey is completed by school administrators or health/physical education staff. Respondents answer questions asking if their school had various policies and programs within the last year at their school, including the following: program implemented to increase walking/biking, improvements to sidewalks, crosswalks, crossing signals, or bike trails, improvements to safety signage around school, school drop-off and pick up policies, existence of school policy to support or encourage walking/biking to school, curriculum taught to encourage walking/biking to school, participation in Walk to School Day, and communications to families about walking/biking. Response options for these items are 0–2 (0 = “No”, 1 = “Yes”, 2 = “I don’t know”). The school policy questionnaire was adapted from similar surveys assessing school-level policies and programs [28,50,51]. In addition, campus improvement plans published by each school district are collected each year of the study and analyzed for mentions of SRTS or related programming. These data are used to assess any prior SRTS or individual school initiatives or policies, as well as secular trends.

#### 2.6.2. Measures: Cohort Study

Child physical activity is measured using accelerometry. ActiGraph wGT3X-BT (ActiGraph, LLC, Pensacola, FL, USA) accelerometers are used to objectively quantify total minutes per day of MVPA among cohort participants. GPS units (Qstarz BT-Q1000XT, Qstarz Intl. Co., Taipei City, Taiwan) are used to record the continuous geographic locations (waypoints) of cohort participants over a seven-day period, matching the accelerometer wear days [52]. Accelerometry and geographic positioning system (GPS) technologies are combined through a time-matched data collection and analysis protocol to objectively assess ACS among study participants [53]. Used together, these devices allow assessment of physical activity per participant, as well as critical contextual factors, such as routes taken to and from school.

In addition to the device-based physical activity measures, parent–child dyads complete surveys that assess psychosocial and behavioral constructs related to ACS and physical activity. The child survey is administered at each of the four time points in the cohort study and has been adapted from the survey previously used in the Texas Childhood Obesity Prevention Policy Evaluation (TCOPPE) study for fourth grade children [28], as well as from the Texas School Physical Activity and Nutrition, CDC Kids-Walk-to-School program, Physical Activity Questionnaire for Older Children, and other surveys [54,55,56,57,58,59,60]. Participants complete the self-reported physical activity measures during the accelerometer/GPS in-person data recovery period for the child, so that the parent–child dyad 7-day recall time frame is consistent.

The parent survey includes questions about ACS, child physical activity, adult physical activity, self-efficacy and outcome expectations about ACS, physical activity knowledge, social cohesion, and social capital. Parents also provide self-reported information on age, gender, race/ethnicity, and socioeconomic status (e.g., educational level of parents) [61,62]. Questions were adapted from the National SRTS parent survey [63], the Urban Hispanic Perceptions of Environment and Activity among Kids (UH-PEAK) study [64], the Neighborhood Environment Walkability Survey (NEWS) [65], the School Physical Activity and Nutrition (SPAN) survey [61], and other surveys with good psychometric properties [55,56,66,67,68].

Key informant interviews are collected from Austin SRTS program participants, as well as from key stakeholders, at baseline and throughout the study to provide additional information about exposure and compliance, as well as narratives for use with decision makers [69]. Key informants (community members, school administrator/teacher, parent, child) selected from 10 cohort schools are interviewed about facilitators and barriers for SRTS, strategies used to address barriers, and additional assistance and resource needs.

#### 2.6.3. Measures: Neighborhood Environment

The Microscale Audit of Pedestrian Streetscapes (MAPS) is being used to collect data on micro-scale built environment features of the surrounding neighborhoods of schools in the cohort study [70,71,72]. For this study, a new version of MAPS adapted to assess SRTS projects (MAPS-SRTS) is being used, which specifically focuses on aspects of routes to school, and includes a section to assess the “school access street segment.” The primary goal of the environmental audit is to assess the streetscape features that are within close proximity to schools. Neighborhood audits were conducted at baseline (2018) and will be conducted again in Year 4.

In addition to the environmental audit, ArcGIS 10.4 (Esri Corporation, Redlands, CA, USA) is being used to integrate spatial and non-spatial data to create a robust geospatial database of relevant neighborhood social and built environment characteristics that may influence ACS and overall physical activity among children. Residential addresses of study participants of the cohort sample, and all study school addresses have been geocoded. The surrounding school neighborhood is defined by a 1.0-mile buffer (sample selection area for cohort participants). GIS data analysis methods [73,74,75,76] were used to construct a series of ‘school neighborhood’ variables, as well as characteristics related to community design. Raw geospatial data (shapefiles, geodatabases) were obtained from public secondary sources, including city, state, and federal governmental agencies (e.g., U.S. census, City of Austin GIS data portal). The 2018 American Community Survey was used to develop baseline GIS variables, including household income, vehicle ownership, and multifamily dwellings [77]. In addition to constructing these GIS variables for the school neighborhood (1.0 mile buffer), upon completion of the study, we will construct the same spatial indicators using sausage buffers around each cohort participant’s route to school, determined by time-matching the accelerometry and GPS data.

#### 2.6.4. Measures: Cost Analysis

Upon completion of the SRTS infrastructure construction, cost data will be provided by the City of Austin, using actual building costs for infrastructure projects at each of the schools, as well as other crucial components such as design and project management costs. In addition to SRTS expenditures, the cost of any other improvements at the schools (e.g., sidewalks, road improvements, etc.) incurred as part of the Mobility Bond will be incorporated into the infrastructure building costs.

### 2.7. Statistical Analysis

Baseline results presented in this paper are from the serial cross-sectional study. School descriptive characteristics are from the 2018–2019 school year from the Texas Education Agency and include mean school enrollment, racial/ethnic distribution, and economically disadvantaged students. GIS-based neighborhood descriptive characteristics use a 1-mile Euclidean buffer with the school address as centroid from the 2018 5-year estimates from the U.S. Census Bureau’s American Community Survey and include connectivity defined as number of three- and four-way intersections, mean household income in dollars, percent households with one or more vehicles, and percent households that are multifamily dwellings. Descriptive characteristics were summarized by study arm, and two-sample independent t-tests were used to assess differences between the Austin SRTS and comparison schools [74,75]. ACS was assessed at the school-level, and the tallies were conducted in Austin SRTS schools prior to infrastructure changes. For each school, the percentage of trips per day made by active travel modes (walking or biking) was averaged across classrooms. The school-level ACS percentage was then averaged across the three weekdays of data collection [28]. ACS was assessed for the total trips to and from school, as well as stratified by trips to or from school. Two-sample independent t-tests were used to determine significant differences in the school-level mean percentage of ACS trips made by active travel modes between Austin SRTS and comparison schools, for total trips, to school trips, and from school trips. To assess differences in the presence of policies and programs by study arm, chi-square tests of independence were used with eight items (walking/biking program, improvements to sidewalks, crosswalks, crossing signals, or bike trails, safety signage, school drop-off and pick up policies, school policy to support walking/biking to school, ACS curriculum, participation in Walk to School Day, and communications to families) from the baseline school health policy survey. All analyses were conducted using R (RStudio version 1.3.959) and the type-I error level was set to 0.05.

## 3. Results

### 3.1. Description of Serial Cross Sectional Study Schools

A total of 94 elementary schools were recruited into the STREETS serial cross-sectional study, 69 of which fall within the City of Austin limits and are included in the Austin SRTS intervention arm (Table 2). Out of the 110 eligible elementary schools in the City of Austin, 69 schools (62.3%) agreed to participate, 10 schools (9.0%) were excluded because the school was slated to close during the study timeline, was undergoing complete school renovation, or had 100% transfer student rate, 15 schools (13.7%) declined, and 16 schools (14.5%) did not respond. At baseline, comparison schools (*n* = 25), on average, had higher enrollment (*p* = 0.02), while the Austin SRTS schools had a significantly higher proportion of Hispanic students (*p* = 0.03), and economically disadvantaged students (*p* < 0.001). There were also significant differences at the neighborhood level. The neighborhoods surrounding Austin SRTS schools had significantly higher connectivity (*p* = 0.002) and a higher percent of households that are multifamily dwellings (*p* < 0.001). The neighborhoods surrounding comparison schools had a higher percent of households with one or more vehicles (*p* < 0.001). There was not a significant difference in the mean household income between the Austin SRTS and comparison school neighborhoods.

### 3.2. Baseline Active Commuting to School

**Figure 2:**
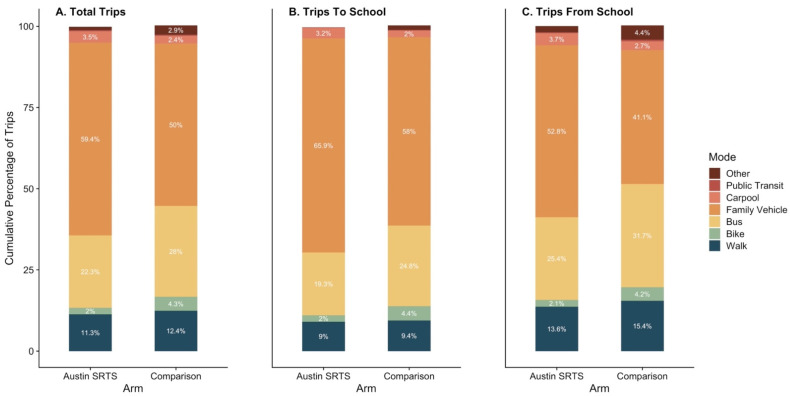
Baseline travel mode to and from school by the Safe TRavel Environment Evaluation in Texas Schools (STREETS) study arm.

Baseline ACS tally data were collected from 85 schools (64 Austin SRTS schools, 21 comparison schools) across the 2018–2019 and 2019–2020 school years before any infrastructure projects were completed at Austin SRTS schools, representing 19,405 third to fifth grade students (ages 8–11 years). The distributions of travel mode of overall trips, trips to school, and trips from school by study arm are shown in Figure 2. Overall, 14.3% (SD = 9.9, range= 0–38.3%) of total trips to and from school were made by walking or biking; 13.7% of children in Austin SRTS schools reported ACS compared to 15.8% of children in comparison schools, *p*-value = 0.43). When stratified, 11.4% (SD = 6.6) of trips to school were made by walking or biking, while 16.6% (SD = 12.2) of trips home from school were made by walking or biking. There were no significant differences in the percent of walking and biking trips made to school (*p*-value = 0.49) or from school (*p*-value = 0.58) between study arms. Most children traveled to and from school by family vehicle, although a significant number also traveled by bus.

### 3.3. Baseline School Policies and Programs

Baseline data on school programs and policies around walking and biking were collected at 76 schools, representing 84% of the total sample (Table 3). The majority (97.4%) reported a school drop-off and pick up policy that includes crossing guards or other elements that encourage safe walking or biking. Less than one-quarter (23.7%) of study schools reported sending communications to families encouraging ACS, while 61.8% of schools had at least one ACS related policy in place. There was a significant difference in the percentage of schools that had walking/biking curriculum taught in every grade, with a higher percentage of Austin infrastructure schools (54.4%) having curricula in place, compared to non-infrastructure schools (15.8%).

## 4. Discussion

STREETS is an ongoing multi-component, multi-level natural experiment study designed to conduct a robust assessment of the population- and individual-level effects of the large-scale implementation of SRTS infrastructure in cities. The overall prevalence of ACS at baseline in the STREETS schools was 14.3%, which is higher than current national estimates [11], but not optimal for child health and to optimize overall physical activity among children [78,79]. Previous work conducted in the Austin area has shown that parental attitudes can be an important determinant in whether a child walks or bikes to school [80,81]. Because elements of the built environment appear to ameliorate parental safety concerns [82], the Austin SRTS efforts to alter travel environments around schools to be more walker and biker friendly can potentially lead to increases in ACS.

Reported policies or programs to promote SRTS in the schools were low, with the exception of school drop-off and pick up policies. SRTS programs have been found to increase ACS in the short term [28], indicating the need to address programmatic efforts along with the built environment when examining ACS [80]. Infrastructure, rather than program, changes are the focus of the STREETS study, so it is important to collect policy and program data as well, so this information can be used in the analysis to control for potential confounding. 

STREETS can be considered a second-generation study, since a robust natural experiment examining both school and individual-level behavioral and health outcomes has not been conducted previously, yet our study builds on past efforts to characterize the effects of SRTS on physical activity and ACS [25,82,83,84,85,86,87]. Unique aspects of STREETS include the use of objective physical activity and location data, the analytic plan, and incorporation of a cost analysis.

Few SRTS studies have focused on objective data [24,25]; most have relied on active commuting tallies or self-report data. In the STREETS study, both SRTS student tallies [35,48], as well as accelerometers are used. ActiGraph monitors, the accelerometers used in this study, have been validated in laboratory and free-living conditions for various age groups, including children [88,89,90]. When used alone, a known limitation of accelerometers is their lack of sensitivity to accurately capture bicycling behaviors [88], an important form of ACS. GPS data will be time-matched with accelerometry data to obtain an objective measure of travel mode, and to identify home-to-school and school-to-home trips of all types (both active and sedentary commuting modes), frequency of ACS, and time spent per trip in active travel modes [53]. The integration of accelerometry and GPS data, along with geocoded participant home and school addresses, will used to map the route that each participant takes to go from home to school in the morning, and from school to home in the afternoon. These data present more complete contextual factors than reported in previous work.

Our analytic plan includes state-of-the-art multi-level modeling that controls for variables such as socio-demographic characteristics of both schools and students. Cohort analyses will include calculation of intracluster correlation coefficient (ICC), and subsequent multi-level modeling that accommodates for individually varying times of participants, individual distances from the child’s residence to the school, and time variant (e.g., GIS and audit-based neighborhood social, food, and built environment measures) and invariant variables (e.g., sex or ethnicity). Cost analysis will determine cost effectiveness using yearly cost per metabolic equivalent (MET) minutes added, which assesses whether the SRTS investments are a good value relative to alternate health investments, such as school-based nutrition programs or smoking cessation [91].

As with other natural experiments, there are risks of bias in this study. First, selection bias may influence outcomes [92,93], especially if there is an inherently unequal distribution of children who live within a walkable distance from the school, or if parents do not let their children walk to school. Although selection bias is difficult to control in natural experiments, it will be addressed through a detailed description of our study samples, as well as our analytic techniques. Bias due to time of measurement can be an issue, especially in school-based studies of outdoor physical activity, where seasonality of measures can affect outcomes. Although some studies have shown significant variation in ACS in spring versus fall [94], these studies are often in places with significant seasonal temperature and climate variability, which is generally not true in the Central Texas area. We will address this by collecting weather-related data each measurement period to use as confounding variables. In addition, we have cross-sectional data on school-level percent of children engaging in ACS, which can be used to compare to any fluctuations observed in the student sample.

In natural experiments there is the possibility of bias due to departures from intended interventions that are beyond the control of the investigators [92]. Even though construction began in 2018 and our study timeline has been designed to allow some flexibility due to construction delays, the length of time to see changes in behavior may be longer than the measurement period, as studies show that initiation of behavioral changes due to environmental interventions may have a lag period [95]. Another potential limitation of this study is that the Austin SRTS program was funded as part of an overall mobility improvement plan for the City of Austin, which includes complementary infrastructure changes to supplement SRTS changes. To address this issue, in addition to data provided by the City of Austin about the types and costs of projects that are constructed around study schools, neighborhood audits and school policy surveys will collect data on any additional infrastructure improvements.

The COVID-19 pandemic occurring during the STREETS study period has provided both challenges and opportunities for the study. Disruptions in school attendance due to the COVID-19 pandemic has affected data collection, but a close partnership between our study team and the City of Austin and participating school districts has allowed for flexibility in data collection to coincide with some of the construction schedules. Additionally, there are potential impacts of the pandemic on participant retention due to high rates of students moving schools. Having objectively-measured MVPA and survey data at baseline allowed us to pivot our data collection during COVID-19 to assess pandemic-related changes in physical activity among children who are learning in a virtual environment rather than in school classrooms. In addition, the quantitative and qualitative data collected will allow us to determine the contextual contributions of ACS to child physical activity during the pandemic, as well as how the pandemic affected ACS in general.

## 5. Conclusions

The STREETS study is a first-of-its-kind natural experiment that has the potential to determine changes in ACS prevalence, and individual-level health-enhancing physical activity (MVPA) in school-age children. Despite some differences in demographic and neighborhood level characteristics, schools in the Austin SRTS study arm did not have a significantly higher proportion of students engaging in ACS than comparison schools at baseline. However, baseline data indicate that Austin SRTS schools have a need for infrastructure changes to optimize ACS, as well as coordinating programmatic efforts to promote walking and biking. Since SRTS programmatic, policy, and infrastructure can increase student ACS [25], it is important for a successful initiative to include all three levels of intervention. Data from the additional study timepoints and measures will indicate the short-term effects of a coordinated SRTS municipal intervention. By providing longitudinal, objective measures of both population-level ACS and individual-level physical activity, this study can serve as a model for designing and implementing robust natural experiment evaluations of the health-related impact of real-world policy interventions. Results from the STREETS study will provide critical evidence on the effectiveness of the use of municipal funding, combined with robust infrastructure audits and project prioritization to inform scale-up and optimal implementation of SRTS programs that can help promote ACS, road safety, and child physical activity in other cities. Schools, neighborhoods, and cities can use the data from this study to inform families of the benefits of infrastructure that supports ACS, advocate for municipal funding, and provide evidence when seeking funding from federal or state transportation programs.

## Figures and Tables

**Table 1:** Safe TRavel Environment Evaluation in Texas Schools (STREETS) study design, measures, and time periods.

Measures	Instruments/Data Source	Data Collection Periods
Hypothesis 1, serial cross-sectional study		
School-level ACS	SRTS student tally measures	Each fall and spring semester
Hypothesis 2, quasi-experimental cohort study		
Child objectively-measured PA, travel mode	Accelerometry, GPS	Third grade (prior to infrastructure), fourth grade, fifth grade fall and spring
Child self-reported PA, ACS, ACS self-efficacy, self-reported sedentary activity	Child survey	Third grade (prior to infrastructure), fourth grade, fifth grade fall and spring
Parent-reported child PA, ACS, neighborhood safety, self-efficacy, knowledge	Parent survey	Third grade (prior to infrastructure), fourth grade, fifth grade fall and spring
School neighborhood environment audits	MAPS-SRTS, GIS-based social and built neighborhood environment	Years 1 and 4
Cost effectiveness component		
Cost of City of Austin SRTS infrastructure changes at schools	City of Austin engineering plans	After infrastructure changes are complete
Policy evaluation, process evaluation, and contextual factors		
Child demographics	Child survey	Baseline for cohort
Parent demographics	Parent survey	Baseline for cohort
School demographics	Data from Texas Education Agency	Fall 2018
Climate	Data from NOAA	All measurement periods
School SRTS programs, other PA-related policies	School policy survey, Campus Improvement Plans	Once per school year

Notes: abbreviations—SRTS, Safe Routes to School; PA, physical activity; TEA, Texas Education Agency, NOAA, GPS, MAPS-SRTS, GIS.

**Table 2:** Descriptive characteristics of the Safe TRavel Environment Evaluation in Texas Schools (STREETS) serial cross sectional study schools.

Descriptive Characteristic	Total *n* = 94 Mean (SD)	Austin SRTS *n* = 69 Mean (SD)	Comparison *n* = 25 Mean (SD)	*p*-Value
School Level Characteristics				
School Enrollment	576.0 (191.4)	550.8 (197.5)	645.6 (156.6)	0.02
Percent Racial/Ethnic Distribution				
Hispanic	53.6 (23.8)	56.3 (25.6)	46.2 (15.9)	0.03
White, non-Hispanic	25.9 (20.6)	23.5 (22.3)	28.7 (14.8)	0.19
African American	9.2 (6.7)	8.5 (7.1)	11.0 (5.4)	0.07
Other	12.3 (10.1)	11.7 (10.8)	14.0 (7.8)	0.25
Percent Economically Disadvantaged Students	53.1 (32.5)	58.7 (34.6)	37.5 (18.5)	<0.001
Neighborhood Level Characteristics				
Connectivity (number of three- and four-way intersections within a 1-mile Euclidean buffer)	247.2 (102.5)	261.2 (104.4)	190.3 (72.3)	0.002
Household income in USD, within a 1-mile Euclidean buffer	76,207.2 (30,243.9)	74,109.6 (32,479.5)	84,720.8 (16,730.8)	0.06
Percent of households with one or more vehicles within a 1-mile Euclidean buffer	94.5 (4.0)	93.8 (4.2)	97.2 (1.8)	<0.001
Percent of households that are multifamily dwellings within a 1-mile Euclidean buffer	30.9 (19.0)	34.3 (18.4)	17.1 (15.0)	<0.001

**Table 3:** Walking and biking school policies and programming from the Safe TRavel Environment Evaluation in Texas Schools (STREETS) school health policy survey.

Policy or Program Reported within the Last Year	Total *n* = 76 *n* (%)	Austin SRTS *n* = 57 *n* (%)	Comparison *n* = 19 *n* (%)	*p*-Value
Program implemented to increase walking/biking in past year	20 (26.3%)	17 (29.8%)	3 (15.7%)	0.36
Improvements to sidewalks, crosswalks, crossing signals, or bike trails in past year	29 (38.2%)	22 (38.6%)	7 (36.%)	0.99
Improvements to safety signage around school in past year	15 (19.7%)	10 (17.5%)	5 (26.1%)	0.62
School drop-off and pick up policy includes use of lane closures, cones, or crossing guards	74 (97.4%)	55 (96.5%)	19 (100%)	0.99
Existence of school policy to support or encourage walking/biking to school	47 (61.8%)	38 (66.7%)	9 (47.4%)	0.22
Curriculum taught to encourage walking/biking to school	34 (44.7%)	31 (54.4%)	3 (15.8%)	0.008
Participated in Walk to School Day in past year	25 (32.9%)	17 (29.8%)	8 (42.1%)	0.48
Communication sent to families about walking/biking	18 (23.7%)	14 (24.6%)	4 (21.0%)	0.99

## Data Availability

The data presented in this study are available upon request from the corresponding author.
